# Oral and parenteral treatment with a third-generation cephalosporin promotes the proliferation of diverse ESBL-producing *Escherichia coli* in the chicken intestinal tract

**DOI:** 10.1128/msphere.00227-25

**Published:** 2025-06-27

**Authors:** Lázaro López, Melany Jumbo, Pamela Mosquera, Gustavo Donoso, Jay Graham, Gabriel Trueba

**Affiliations:** 1 Instituto de Microbiología, Colegio de Ciencias Biológicas y Ambientales, Universidad San Francisco de Quito 603002 , Quito, Ecuador; 2 Ingeniería en Biotecnología, Facultad de Ingenierías y Ciencias Aplicadas, Universidad de las Américas 126422, Quito, Ecuador; 3 Population Health and Pathobiology, North Carolina State University at Raleigh 6798, Raleigh, North Carolina, USA; 4 Environmental Health Sciences Division, University of California 218536 , Berkeley, California, USA; University of Michigan-Ann Arbor, Ann Arbor, Michigan, USA

**Keywords:** *Escherichia coli*, *bla*_CTX-M_, antimicrobial resistance, ceftriaxone, cephalosporin, poultry, chicken, *bla*_CTX-M-55_, third-generation cephalosporin, antibiotic resistance

## Abstract

The global rise of antimicrobial resistance is a major public health threat, with *Escherichia coli* facilitating the spread of extended-spectrum beta-lactamase (ESBL) genes like *bla*_CTX-M_, which confer resistance to third-generation cephalosporins (3GCs). This study examines the impact of 3GC treatment on resistant *E. coli* clones and horizontal gene transfer (HGT) of ESBL genes in broiler chickens in Quito, Ecuador. Fifteen-day-old Ross broilers were divided into three groups: oral ceftriaxone (100 mg/kg), parenteral ceftriaxone (100 mg/kg intramuscular), and control (no treatment). The study included three phases: baseline, antimicrobial administration (5 days), and recovery (15 days). Fecal cultures on McConkey agar, with and without ceftriaxone (2 µg/mL), measured the ratio of 3GC-resistant lactose fermenters. Regardless of the administration route, ceftriaxone significantly increased resistant coliforms (>80%). Five *E. coli* colonies per animal and time point were analyzed using single-gene typing, with clonal candidates subjected to whole-genome sequencing. Clonal analysis revealed high genetic diversity, averaging three distinct clones per animal. A unique lineage (H34) emerged exclusively during treatment, and new clones appeared post-treatment. The *bla*_CTX-M-55_ variant was the most abundant ESBL gene, persisting despite fluctuations in other *bla*_CTX-M_ variants. Comparative plasmid analysis suggested *bla*_CTX-M-55_ HGT, as plasmids were identified in two genetically distinct *E. coli* isolates from the same host. Most plasmids belonged to IncFII, with IncX1 and IncN also present. These findings highlight how 3GC treatments rapidly impact ESBL-producing *E. coli* diversity in the intestine.

**IMPORTANCE:**

The global rise of antimicrobial resistance (AMR) poses a critical public health challenge, with *Escherichia coli* playing a central role in the spread of extended-spectrum beta-lactamase (ESBL) genes like *bla*_CTX-M_, which confer resistance to third-generation cephalosporins (3GCs). This study highlights the significant impact of 3GC treatment on the frequency and diversity of 3GC-resistant *E. coli* clones and horizontal gene transfer of ESBL genes in the intestinal microbiota of broiler chickens. Understanding how antimicrobial treatments drive resistance dynamics in animal populations is crucial for developing strategies to mitigate AMR in both human and veterinary settings.

## INTRODUCTION

The global rise in antimicrobial resistance (AMR) represents a critical challenge for both human and veterinary medicine. AMR is a pressing public health threat that claimed an estimated 1.27 million lives worldwide and was associated with nearly 5 million deaths in 2019 (1).

Antimicrobial resistance genes are constantly transmitted in the intestine via horizontal gene transfer (HGT) (2); moreover, conjugation of some plasmids, carrying extended-spectrum beta-lactamase (ESBL) genes, increases under antimicrobial pressure (3). Among the organisms of concern, *Escherichia coli* is the most common facultative anaerobe in the intestine of warm-blooded animals (4). This bacterium is primarily a commensal that is frequently implicated in the transmission of resistance genes to pathogenic bacteria (5).

One of the most concerning AMR genes that are being transferred by *E. coli* is the ESBL gene, such as the *bla*_CTX-M_ family, which confers resistance to third-generation cephalosporins (3GCs). These genes have been increasingly disseminated since the 1990s to today and are the most dominant 3GC resistance genes (6–9).

It has been observed that humans and domestic animals carry different clones of *E. coli* in the intestine at any time (10–12), and some of these clones may have 3GC resistance genes. Additionally, *E. coli* clones in the intestine often have a high rate of turnover (12–14). This study set out to understand how treatment with 3GCs alters the frequency of naturally occurring 3GC-resistant *E. coli* clones in the intestine and whether the treatment route (oral vs parenteral) has differential impacts on levels of resistance in the intestines. In this study, we used commercially sold chickens in Quito-Ecuador, which are known to naturally carry 3GC-resistant *E. coli* (15), to study the effect of 3GC administration over the population of 3GC-resistant *E. coli* in the chicken intestinal tract. We used plasmid sequencing to investigate the HGT of some of these resistance genes within the chicken intestinal tract.

## MATERIALS AND METHODS

### Experimental design and animal groups

**Fig 1:**
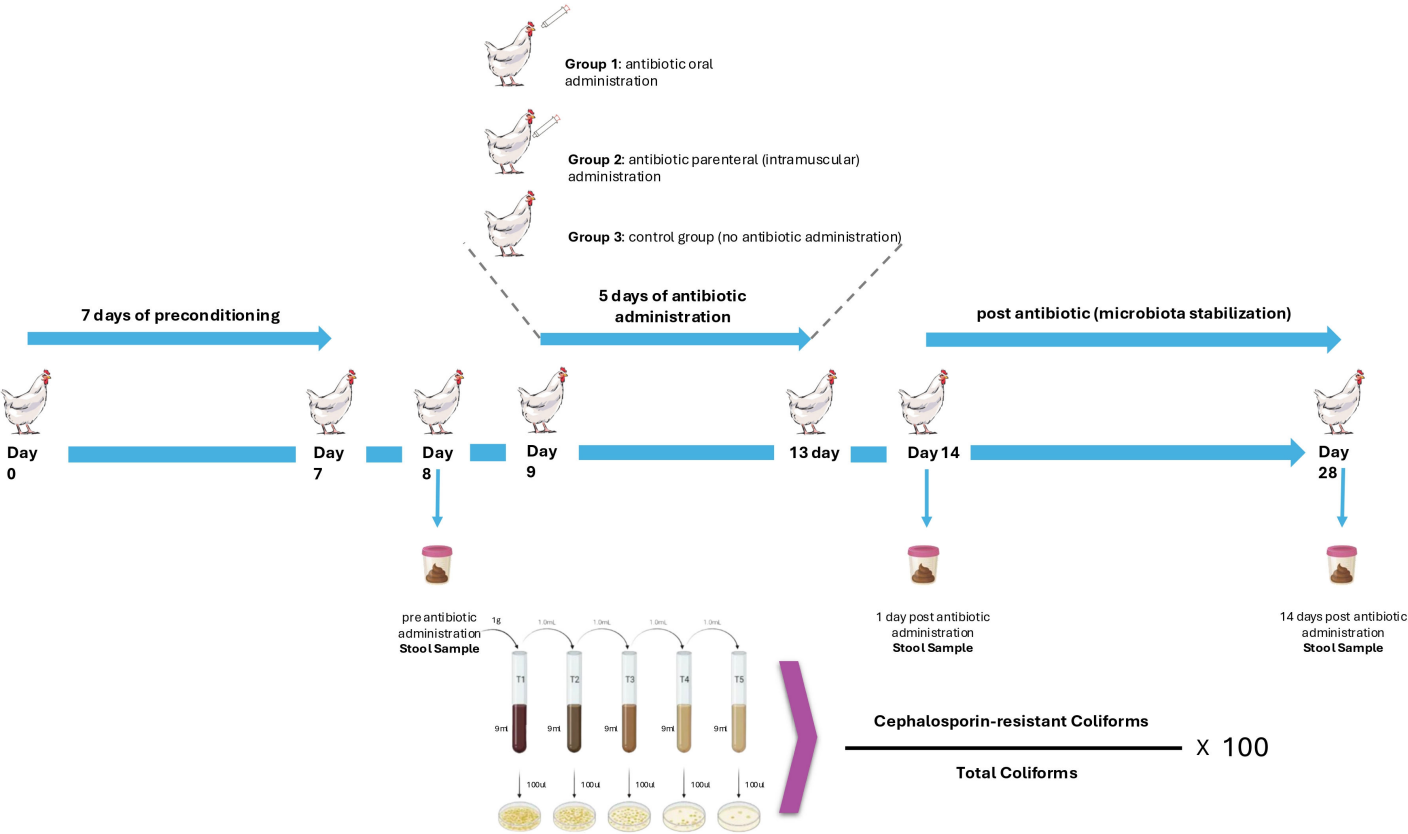
Diagram of the experimental design, highlighting sampling time points: (i) before ceftriaxone administration, (ii) 1 day after completing a 5-day ceftriaxone treatment, and (iii) 15 days after treatment.

The study was conducted using Ross broiler chickens (15 days old) that were housed in a controlled environment. Twenty-one chickens were divided into three groups (seven in each group): (i) oral ceftriaxone group: received ceftriaxone orally at 100 mg/kg dose (16); (ii) parenteral ceftriaxone group: administered ceftriaxone intramuscularly at same dose; and (iii) control group: received no antimicrobial treatment. The experiment spanned three phases: pre-administration (baseline), antimicrobial administration (5 days), and post-administration recovery (15 days; Fig. 1). Chickens were placed in spacious cages according to the poultry handling methodology proposed by the European Union (17). Each cage had a minimum height of 35 cm and an area of 750 cm^2^ per chicken, with bars spaced no more than 2 cm by 15 cm to prevent leg injuries. The cages were designed to allow droppings to fall through to a plastic tray, avoiding accumulation on the bars or the chickens’ legs. Feed and water were provided *ad libitum* using standardized feeders and drinkers. The chickens were fed ground corn free of antimicrobials and growth promoters. Each group was housed in a 1 m^2^ cage, with seven chickens together.

### Isolation and single-gene typing of *E. coli* isolates

Fecal samples were collected at each time point and processed to isolate coliform bacteria using MacConkey Lactose Agar (MKL) supplemented with 2 µg/mL of ceftriaxone, a 3GC, following standard protocols for selective isolation (18).

To assess the ratio of third-generation cephalosporin-resistant coliform bacteria in the intestinal microbiota, fecal samples were serially diluted in 0.9% sterile saline and spread-plated onto both MKL without antimicrobials and MKL supplemented with ceftriaxone (2 µg/mL). The initial dilution was prepared by weighing 1 g of feces and diluting it in 9 mL of sterile saline, resulting in a 10^−1^ dilution, followed by subsequent serial dilutions. Each dilution was plated in triplicate on both antimicrobial-free and antimicrobial-supplemented MKL. Plates were incubated at 37°C for 24 hours, and the ratio was calculated by determining the number of resistant bacterial colonies (CFU/mL) grown on antimicrobial-supplemented medium over the total bacterial colony count (CFU/mL) grown on antimicrobial-free multiplied.

The numerically dominant *E. coli* was identified by randomly selecting five *E. coli* colonies per sample (19). These lactose-fermenting colonies were then re-streaked on Chromocult Coliform Agar (Merck, Germany) to confirm a typical *E. coli* phenotype based on β-glucuronidase activity (4-methylumbelliferyl-β-D-glucuronide [MUG]-positive). We also amplified and sequenced the *E. coli fliC* gene (see below) from all these isolates. From each sample, five 3GC-resistant colonies with the characteristic *E. coli* phenotype were selected for additional analysis.

A total of 105 3GC-resistant *E. coli* isolates were screened for clonality using the *fliC* gene (encoding the *E. coli* flagellin protein) (14). This gene was amplified as previously described (20), and PCR products were purified and sequenced by Macrogen (Seoul, South Korea) using Sanger sequencing technology. Using SerotypeFinder 2.0, we determined the H alleles of each *E. coli* strain.

We used a PCR protocol to amplify 3GC resistance genes (*bla*_CTX-M_, *bla*_TEM_, *bla*_SHV_, and *bla*_OXA_) (21) and found that the *bla*_CTX-M_ gene family was responsible for the resistance in all of the isolates. The PCR products were sequenced by Macrogen (Seoul, South Korea) as described above, and the resulting sequences were analyzed using ResFinder 4.1 (22) and the NCBI Bacterial Antimicrobial Resistance Reference Gene Database (23) for subtype identification.

### Extraction of genomic and plasmid DNA

Some suspected *E. coli* clones and isolates sharing the same *bla*_CTX-M_ gene in the same chicken were whole genome sequenced to investigate horizontal gene transfer. Total DNA was extracted from bacterial isolates using the DNeasy Blood and Tissue Kit (Qiagen, Hilden, Germany) following the manufacturer’s standard protocol for bacterial isolates. This method consistently yields high-quality genomic DNA suitable for molecular analyses. Plasmid DNA was isolated using the ZymoPURE II Plasmid Midiprep Kit (Zymo Research, Irvine, CA, USA) according to the manufacturer’s instructions. DNA concentration and purity were measured with a NanoDrop spectrophotometer (Thermo Fisher Scientific, Waltham, MA, USA) and a Qubit 4 Fluorometer (Thermo Fisher Scientific, Waltham, MA, USA), allowing for precise quantification of both total genomic and plasmid DNA.

### Whole-genome sequencing and comparative analysis

Whole-genome sequencing (WGS) was performed on seven isolates that shared the same *fli*C gene to confirm clonality (E016, E031, E033, E035, E070, E102, and E104). Libraries were prepared using the Ligation sequencing gDNA - Native Barcoding Kit 24 V14 SQK-NBD114.24 (Oxford Nanopore Technologies, Oxford, UK) and sequenced on the Oxford Nanopore GridION platform, flow cell (R10.4.1), following the manufacturer’s protocols.

We performed *de novo* assembly of the raw reads, which were first quality checked using NanoPlot, filtered using Filtlong, and subsequently assembled using Trycycler (v0.5.5), which integrates assemblies generated with Flye (v2.9), Minimap2 (v2.26), and Raven (v1.8.1) to produce high-quality consensus assemblies (24). Genome annotation was performed using Prokka (v1.14.6) and Bakta (v1.7), ensuring accurate identification of coding sequences, functional genes, and genomic features (25, 26). Genome alignments were conducted using Minimap2 and analyzed in Geneious Prime (Biomatters, Auckland, New Zealand) to assess structural variations and conserved regions (27). Summary statistics of the sequencing data, including read length, coverage, and quality metrics, are presented in Table SII.

Single nucleotide polymorphisms (SNPs) were identified using Snippy (v4.6.0), with isolate E016 as the reference genome for comparative analysis. To further classify the bacterial genomes, multilocus sequence typing and core multilocus sequence typing were performed to determine sequence types (STs) and core sequence types (cSTs), respectively, providing insights into the genetic relationships and evolutionary patterns of the isolates (28).

### Plasmid characterization and comparative analysis

Plasmid sequences were annotated to identify replicon types, resistance genes, and insertion sequences using PlasmidFinder (v2.1), ResFinder (v4.1), and ISFinder databases, respectively (29–31). Plasmid multilocus sequence typing was performed to classify plasmid sequence types, providing insights into their diversity and evolutionary history (29). Comparative sequence alignments of plasmids were performed using MAFFT (v7.475) and Geneious Prime (Biomatters, Auckland, New Zealand), enabling the detection of structural variations, resistance cassettes, and other genomic features associated with antimicrobial resistance (32).

### Statistical analysis

Data were analyzed using one-way analysis of variance followed by post-hoc Tukey’s test for multiple comparisons to assess significant differences in bacterial resistance frequencies between the treatment groups (oral, parenteral, and control). For the analysis of changes over time, a linear mixed-effects model was fitted using restricted maximum likelihood. A significance level of *P* < 0.05 was considered statistically significant. All statistical analyses and data visualizations were performed using GraphPad Prism (version 10), and the linear mixed model was implemented using the lme4 and lmerTest packages in R.

## RESULTS

### Ceftriaxone administration route effect

**Fig 2:**
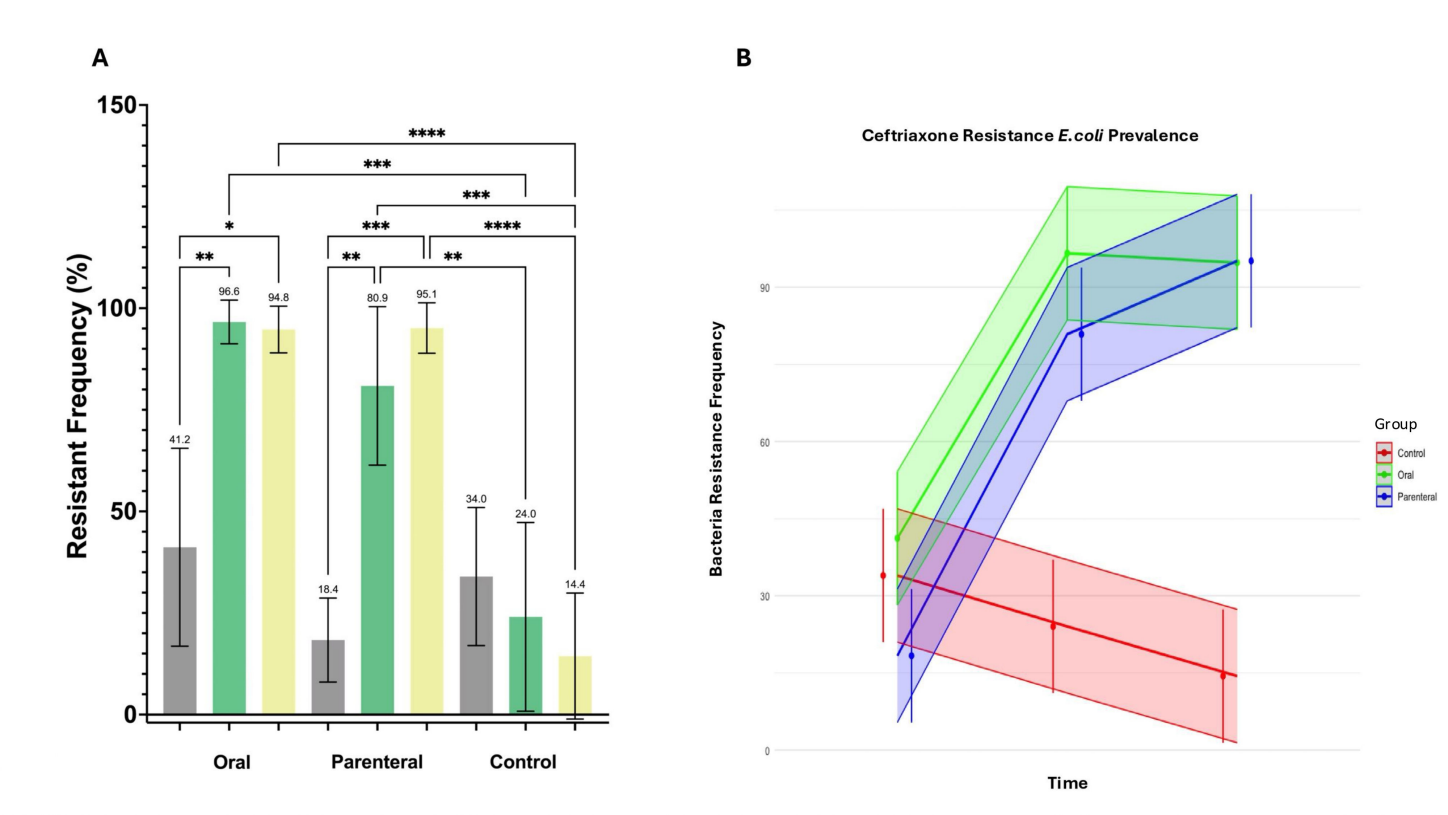
*E. coli* ceftriaxone resistance ratio: (**A**) represents the *E. coli* resistance frequency by treatment group and time, and bars represent the mean and 95% confidence intervals. Above each bar, the exact value of the mean frequency of each one is numbered. Lines represent Kruskal-Wallis tests with an uncorrected Dunn’s multiple comparisons post hoc test and a 95% confidence interval. Asterisks indicate differences between bars showing statistical significance (*P* ≤ 0.05). The number of asterisks represents different *P* values: <0.05 (*), <0.005 (**), <0.0005 (***), and <0.0001(****). (**B**) represents the mixed effect model of the data where the thick lines represent the trend in frequencies of *E. coli* resistant to ceftriaxone and the shaded lines the 95% confidence interval of the data.

The routes of ceftriaxone administration (oral and parenteral) led to an increase of ceftriaxone-resistant *E. coli* in chickens’ intestines (Fig. 2). Interestingly, the 3GC-resistant *E. coli* ratio was high even before the treatment, with a total mean of 31.16% ± 4.59%, with some chickens showing up to 60% of coliform 3GC resistance. Five days of ceftriaxone administration caused an increase in resistance to 96.61% ± 2.19% of the intestinal coliforms in the oral route and 80.88% ± 7.97% in the intramuscular route. The resistance rate remained elevated, 94.77% ± 2.35% and 95.13% + 2.54%, in the animals receiving orally or intramuscularly, 15 days after the ceftriaxone administration was ended. This indicates that the majority of *E. coli* in the intestinal tract of chickens continue to be resistant following antimicrobial administration, irrespective of the route of delivery. The comparison between administration routes oral or parenteral did not show any statistically significant results (*P* value of 0.459 and 0.924, respectively). In control animals with no antimicrobial administration, the resistance rate decreased to 24.04% ± 9.49%, suggesting that the effects found in the groups receiving antimicrobials were a result of this and not some other uncontrolled factor.

### Population dynamics of ceftriaxone-resistant intestinal *E. coli* in the oral administration group

**Fig 3:**
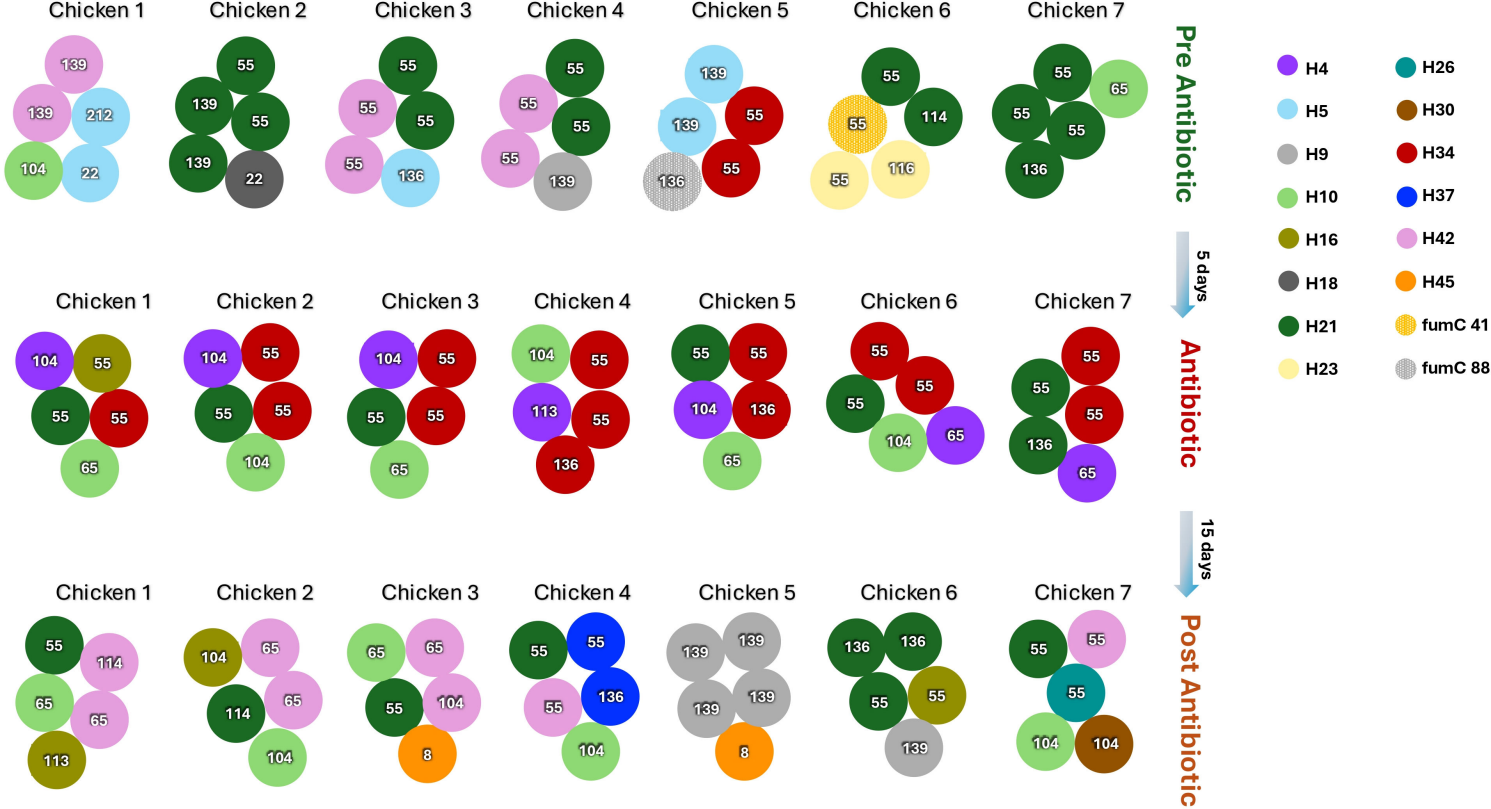
Diagram illustrating the dynamics of ceftriaxone-resistant intestinal *E. coli* populations in the oral antimicrobial administration group. Each circle represents an *E. coli* isolate, with the color of the circle indicating the *fliC* gene allele, and the numbers inside the circle correspond to the *bla*_CTX-M_ gene variant.

Single gene typing revealed significant clonal diversity in ceftriaxone-resistant *E. coli* isolates, with an average of three different clones out of five isolates obtained per animal fecal sample (mean = 3.43 ± 0.19) and ranging from two to five different clones in different animals. The diversity of clones was similar during treatment with an average of 3 (mean = 3.86 ± 0.26) ranging from 3/5 to 5/5 and a post-treatment average of 3 (mean = 3.71 ± 0.36) ranging from 2/5 to 5/5 (Fig. 3). Surprisingly, we did not observe any proliferation of a 3GC-resistant clone detected prior to the treatment. A distinct lineage (H34 = red) was observed exclusively during the antimicrobial administration period. After ceftriaxone treatment, new isolates emerged: H45, H26, H37 (Fig. 3).

### Diversity of *bla*_CTX-M_ gene

All ceftriaxone-resistant isolates harbored *bla*_CTX-M_ genes. Screening for *bla*_TEM_, *bla*_SHV_, and *bla*_OXA_ genes was also performed; these genes were not detected except in a few cases where *bla*_TEM_ was found alongside *bla*_CTX-M_. We found a total of 11 *bla*_CTX-M_ gene variants in all the animals. *bla*_CTX-M-55_ was the most abundant *bla*_CTX-M_ variant (47/105), followed by *bla*_CTX-M-104_ (14/105), and *bla*_CTX-M-65_ and *bla*_CTX-M-139_ (11/105, both). Upon analyzing the diversity and abundance of *bla*_CTX-M_ variants over time, we observed a total of nine allelic variants (ranging from 2 to 5 in the five isolates obtained per animal fecal sample) before treatment, and a total of five allelic variants during treatment ranging from 3/5 to 4/5 per animal fecal sample (Fig. 3). Despite these fluctuations, *bla*_CTX-M-55_ remained the most abundant variant throughout the study; initially, it accounted for more than 50% of the population, maintaining this proportion during antimicrobial treatment, before dropping to nearly 30% by the end of the study. Of the total 35 isolates (five per chicken from seven chickens at each time point), variants *bla*_CTX-M 65_ and *bla*_CTX-M 104_ accounted for only 1% of the isolates before treatment. Their frequency increased to 20% during treatment and stabilized at 17% by the end of the study.

### Features of plasmids carrying the *bla*_CTX-M-55_ variant gene

To determine horizontal gene transfer, we sequenced the plasmids of seemingly different clones (single gene typing) with the same *bla*_CTX-M_ allele in the intestine of one animal at one time point (isolates E031, E033, E035, E070, E102, and E104 in animal 7; isolates E016 and E018 in animal 4; and isolates E022, E023, E056, and E057 in animal 5). Comparative analysis of plasmids showed that most of the plasmids were different; however, plasmids carrying *bla*_CTX-M-55_ were identical in two genetically different isolates, E070 and E102. Both isolates were obtained from the same host (chicken), with E070 isolated 15 days before E102, which suggests a possible horizontal plasmid transfer (Fig. S2A); however, we were not able to demonstrate conjugative capabilities in these plasmids because they lack some of the *tra* genes (data not shown).

Most of the plasmids belonged to the IncFII incompatibility group (pHN7A8); however, we identified plasmids with IncX1 and IncN replicons and some combinations of IncFII and IncX1 (Table 1; Fig. S2). Three distinct genetic environments (brackets) associated with *bla*_CTX-M-55_ were identified, with brackets 1 and 2 being the most prevalent (Fig. S1). In *E. coli* strain E067, two 3GC-resistant plasmids were detected: one carrying bracket 3 and another containing a duplicated bracket 2 flanking bracket 3 (Fig. S1D). This last strain exhibited a complex plasmid structure with duplication of bracket 2 and a single copy of bracket 3, resulting in an increased number of *bla*_CTX-M-55_ copies.

**TABLE 1:** Genomic characteristics and variations of *E. coli* isolates subjected to WGS^*a*^

Sample_name	cgST	ST	*fliC* allele	Genome length (bp)	SNPs	Indels	Complex	
E016	39,108	2,505	H21	4,715,929	Refseq	Refseq	Refseq	
E031	39,108	2,505	H21	4,715,929	3	–	–	
E033	39,108	2,505	H21	4,716,800	3	1	1	872 bp insertion
E035	39,108	2,505	H21	4,702,598	2	–	1	13,331 bp deletion
E070	39,108	2,505	H21	4,715,928	2	1	–	–
E104	39,108	2,505	H21	4,714,593	2	–	1	1,336 bp deletion
E102	119,558	648	H42	5,172,204	47,994	53	N/A	N/A

^*a*^ –, no data; N/A, not applicable.

### Whole-genome sequences of *E. coli* isolates H21 lineage harboring IncFII(pHN7A8) plasmids carrying *bla*_CTX-M-55_

**Fig 4:**
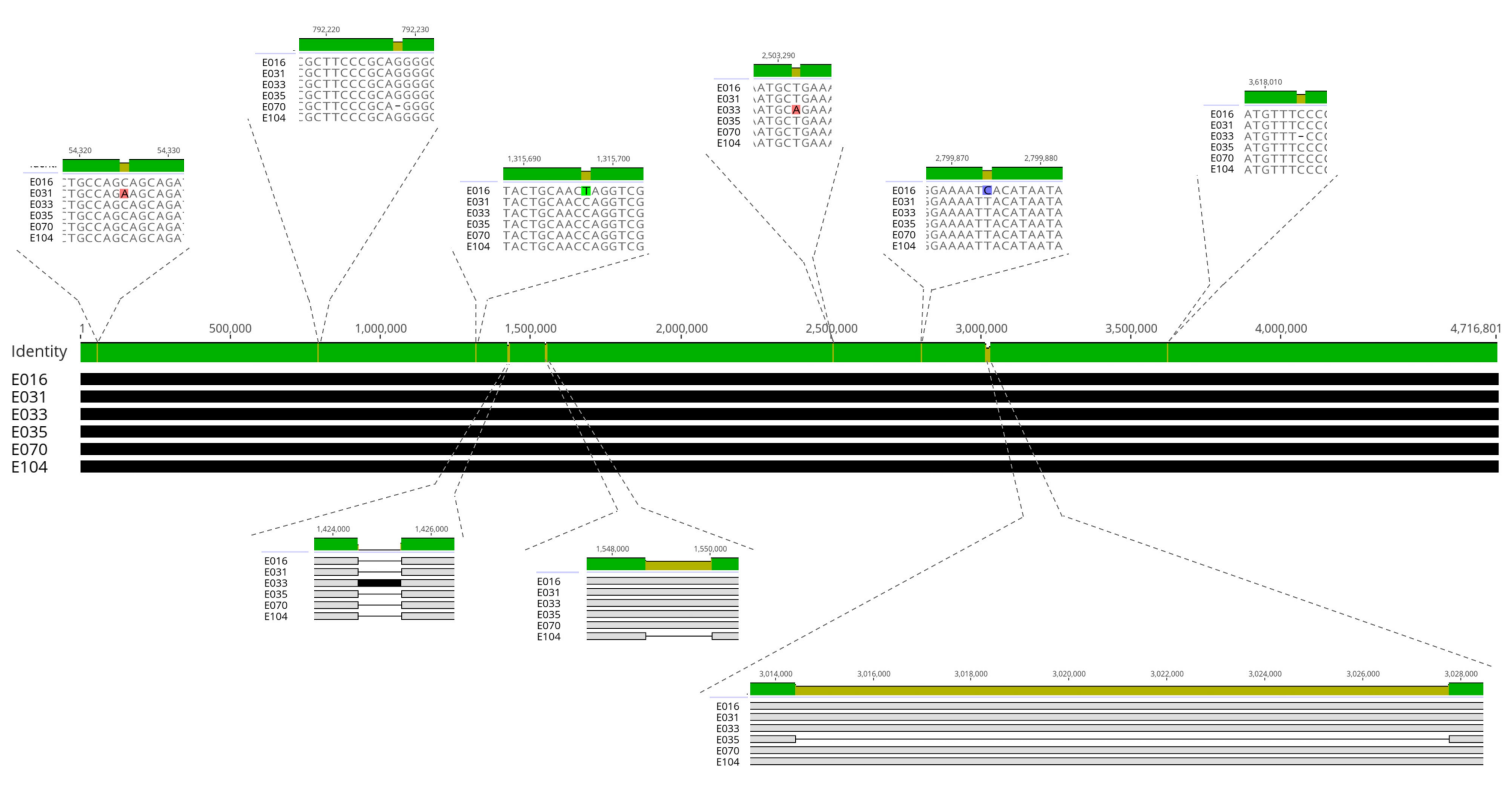
Whole-genome alignment of six *E. coli* isolates carrying the *fliC* H21 allele.

Six isolates with identical *fliC* gene sequence were selected for whole-genome sequencing: E016, E031, E033, E035, E070, E104, and E102. We also sequenced isolate E102, which belongs to a different lineage but harbors the same plasmid identified in isolate E070 (Table 1). The isolates carried the same *fliC* H21 allele and shared similar genome lengths, ranging from 4,704,929 bp to 4,716,800 bp. Comparative WGS analysis of isolates E070 and E102 confirmed that they were distinct *E. coli* clones, as they displayed different cSTs and STs. This finding indicates a potential horizontal transfer event between isolates E070 and E102, probably within the intestine of this chicken (both isolates were obtained from the same host chicken), with isolate E070 being isolated 15 days prior to isolate E102. Additionally, genomic analysis of the six other isolates (E016, E031, E033, E035, E070, and E104) confirmed that they represent variants of the same *E. coli* clone (2–3 SNPs). Despite E016 originating in a different chicken than the others, all six isolates shared the same core ST and ST, and their alignment (Fig. 4) showed high genetic similarity. To further investigate, SNIPPY was employed using isolate E016 as the reference genome. The results revealed that these isolates differed by only 2–3 SNPs. However, isolates E033, E035, and E104 also exhibited insertions and deletions (indels). Two single base-pair (1 bp) deletions were identified in isolates E033 and E104, respectively. Additionally, three complex indel events were observed in these three isolates. Complex indels were defined as insertions or deletions greater than 100 bp (Table 1). In contrast, isolate E102, which belonged to a different lineage (H42), had a significantly larger genome (5,172,204 bp) and exhibited 47,994 SNPs and 53 indels.

We also annotated the whole genomes of the six *E. coli* isolates of the H21 lineage using Prokka. Comparing the regions with SNPs and both simple and complex indels, we observed that isolate E016, originating from chicken 4, differed from the other five isolates, which were derived from chicken 7, by only two SNPs.

## DISCUSSION

As expected, the administration of ceftriaxone to the 14 chickens, both orally (7) and intramuscularly (7), resulted in a significant increase in the 3GC-resistant *E. coli* ratio in the intestines of treated chickens. During the treatment, the resistance levels reached 96.61% in the oral group and 80.88% in the intramuscular group, suggesting that antimicrobial exposure facilitates the survival and proliferation of resistant clones. Before treatment, the baseline 3GC-resistant *E. coli* ratio averaged 31.16% across the 21 chickens, with a range from 7.25% to 86.56%, indicating that substantial resistance was present in the intestinal microbiota even before the antimicrobial intervention.

Genotypic and phenotypic analyses of *E. coli* isolates revealed substantial diversity, including 16 distinct lineages and 11 variants of the *bla*_CTX-M_ gene, further corroborating the clonal and gene variability associated with antimicrobial resistance. We expected that some 3GC-resistant *E. coli*, already present in high numbers in the intestine before treatment, would survive and proliferate during the treatment. Instead, we found that in each chicken, new *E. coli* clones seemed to outnumber the previous ones during treatment; for example, *E. coli* clones H21 (14/35) and H42 (6/35) were more prevalent before treatment in chickens 2 and 7. However, *E. coli* clones H34 (14/35) and H4 and H21 (both 7/35) were more prevalent during treatment in chickens 2, 3, 4, 5, and 6, and H21 and H42 (both 8/35) after treatment in chickens 1, 2, 3, and 6. This observation indicates that some 3GC-resistant *E. coli* clones are more likely to dominate during antimicrobial treatment, whereas other 3GC-resistant *E. coli* clones dominate in the absence of an antimicrobial (Fig. 3). These results may indicate that some *E. coli* clones may better tolerate (have better fitness) a plasmid without antimicrobial pressure (plasmid as a molecular parasite), whereas other clones do better under antimicrobial pressure, which is consistent with previous observations (33–37). The expansion and subsequent disappearance of the H34 clone, during and after ceftriaxone treatment, may indicate that some bacterial clones display different fitness costs with different plasmids; some plasmids carrying *bla*_CTX-M_ genes improve the bacterial fitness in the absence of ceftriaxone (33), whereas other plasmids may provide better fitness (to certain bacterial clones) in the presence of ceftriaxone (38, 39).

The evidence presented here suggests that the proliferation of diverse 3GC-resistant clones may be a more important mechanism for promoting 3GC resistance during and after antimicrobial treatment. However, we detected a potential HGT in one case during antimicrobial treatment: the same plasmid was present in two distinct *E. coli* lineages in the same animal, as seen with plasmid pE070 in E070 (cST 39108) and E102 (cST 119558). Previous reports suggest that antimicrobial treatment increases the horizontal transmission of ESBL genes (3, 40–46).

The predominance of *bla*_CTX-M-55_ in the isolates highlights the role of plasmid-mediated resistance, especially associated with IncFII (pHN7A8) plasmids. Such plasmids are known to facilitate horizontal gene transfer between bacterial isolates, thereby enhancing the spread of resistance genes across bacterial populations (47, 48). Interestingly, the genetic environment of *bla*_CTX-M-55_ (bracket 2) had been previously reported in Ecuador in 2018 in commensal *E. coli* isolated from children, dogs, and chickens, as well as in a uropathogenic *E. coli* strain in 2014 (49, 50). Although the bracket identified in our study shares the same structural organization, it exhibits eight SNPs: one base substitution and seven single-base insertions (Fig. S1). This suggests a close evolutionary relationship and a likely descent from a bracket previously identified in Ecuador. Furthermore, the observed variability in the Ecuadorian brackets indicates an ongoing diversification process, potentially driven by selective pressure from the use of ceftriaxone or other antimicrobials in poultry production. The presence of a complex plasmid structure in strain E067, consisting of a duplicated bracket 2 and a single copy of bracket 3, indicates a genomic rearrangement that increases the number of *bla*_CTX-M-55_ copies. This arrangement suggests that under antimicrobial pressure, the transposable element IS*26* may have promoted the duplication of the *bla*_CTX-M-55_, which is something that has been increasingly reported in recent years (51–54). This IS26-mediated amplification likely leads to higher expression of the ESBL, enhancing bacterial survival under high ceftriaxone concentrations (55).

Interestingly, the resistance rate remained at the same level after 15 days post-treatment, reinforcing the idea that antimicrobial exposure has long-term effects on the intestinal resistome. This observation aligns with previous studies that have documented the persistence of *E. coli* resistant to 3GCs long after the cessation of antimicrobial treatment (56). For example, the persistence of resistance in poultry after ceftiofur treatment has been well documented in previous studies by Liao et al. (57).

The results of this study also provide valuable insights into the dynamics of antimicrobial resistance in poultry production. The prolonged persistence of resistance after ceftriaxone treatment, along with the role of mobile genetic elements in spreading resistance genes, highlights the risks associated with the use of antimicrobials.

The lack of significant differences between the oral and intramuscular routes of ceftriaxone administration suggests that the route of administration does not influence the development of resistance as much as the presence of the antimicrobial resistance itself. These results are consistent with the findings of Seo et al. (56) and others who have observed similar resistance dynamics irrespective of the route of administration (56–58).

In the control group, which did not receive antimicrobials, resistance levels remained significantly lower, reinforcing the notion that ceftriaxone administration was the primary factor driving the increase in *E. coli* resistance. This highlights the importance of responsible antimicrobial use in agricultural settings, as unnecessary or improper use of antimicrobials can contribute to the selection of resistant bacteria in the gut microbiota, ultimately leading to the spread of resistance genes (59). Such findings also provide a crucial understanding of the risks associated with antimicrobial use in poultry farming, as resistant isolates could be transmitted to humans through the food chain or via environmental contamination (60, 61).

This study showed an unexpected genetic complexity of antimicrobial resistance in the intestine during and after 3GC treatment, involving resistant clone proliferation, HGT, and possibly AMR gene duplications. Studies like this offer a unique insight into the complex and dynamic interactions between 3GC resistance genes, plasmids, transposons, and *E. coli* clones during antimicrobial treatment.

## Data Availability

Accession numbers are as follows: BioProject PRJNA1243555; BioSample SAMN47625275, SAMN47625276, SAMN47625277, SAMN47625278, SAMN47625279, SAMN47625280, SAMN47625281, SAMN47629383, SAMN47629384, SAMN47629385, SAMN47629386, SAMN47629387, SAMN47629388, SAMN47629389, SAMN47629390, SAMN47629391, SAMN47629392, SAMN47629393, SAMN47629394, and SAMN47629395.

## SUPPLEMENTAL MATERIAL

Supplemental Material msphere.00227-25-s0001.pdf Supplemental figures and tables.

ASM does not own the copyrights to Supplemental Material that may be linked to, or accessed through, an article. The authors have granted ASM a non-exclusive, world-wide license to publish the Supplemental Material files. Please contact the corresponding author directly for reuse.
