# Supplementary material for: Oral and parenteral treatment with a third-generation cephalosporin promotes the proliferation of diverse ESBL-producing Escherichia coli in the chicken intestinal tract
Source: mSphere. 2025 Jun 27;10(7):e00227-25. doi: 10.1128/msphere.00227-25 (PMC12306158; doi:10.1128/msphere.00227-25)
Supplement: Supplemental Material — Supplemental figures and tables. [file msphere.00227-25-s0001.pdf]

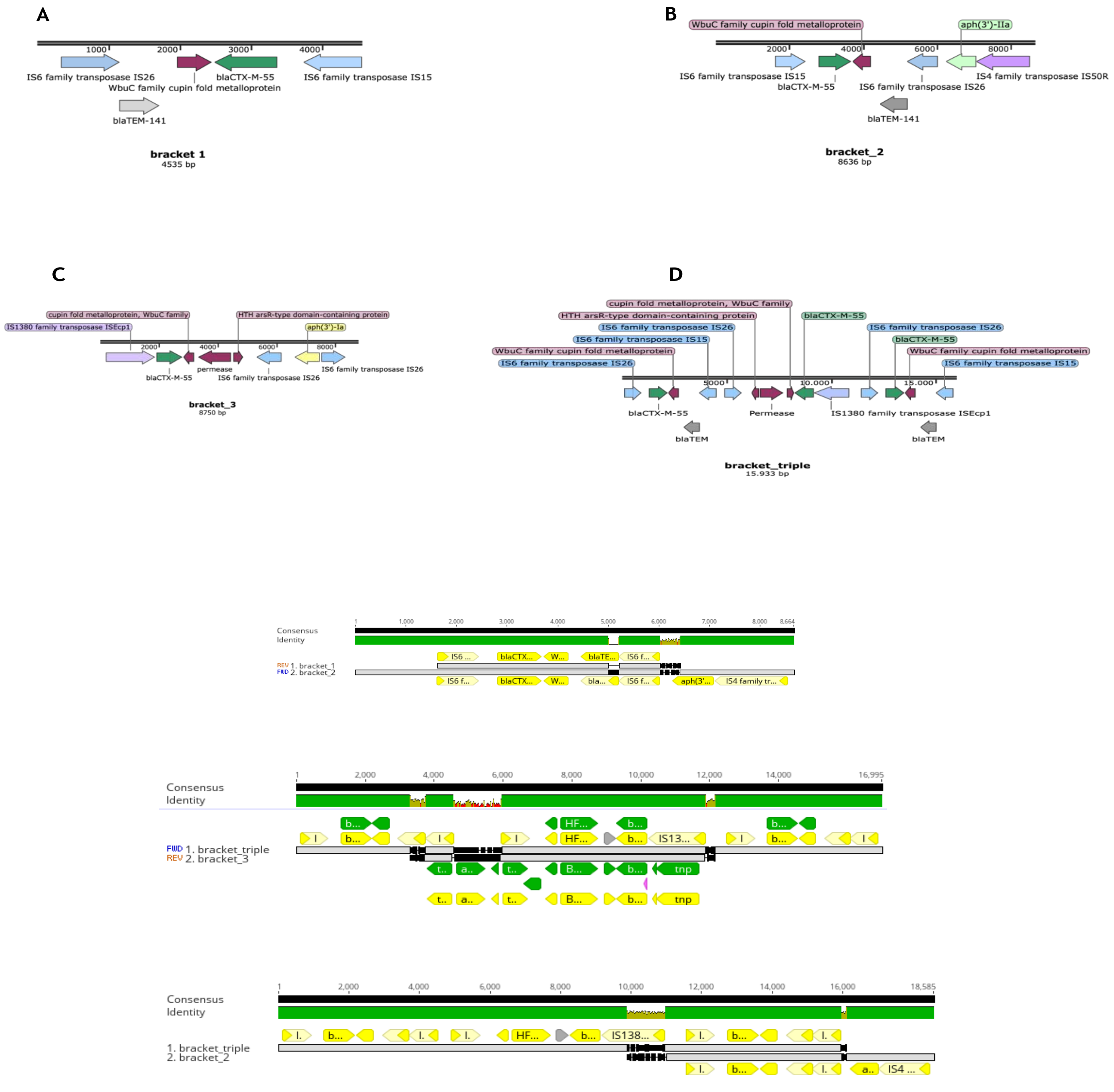

Supplementary Figure 1. Annotation Diagram of Bracket 1 (**A**), Bracket 2 (**B**), Bracket 3 (**C**), and Bracket_triple (**D**) and their paired alignment

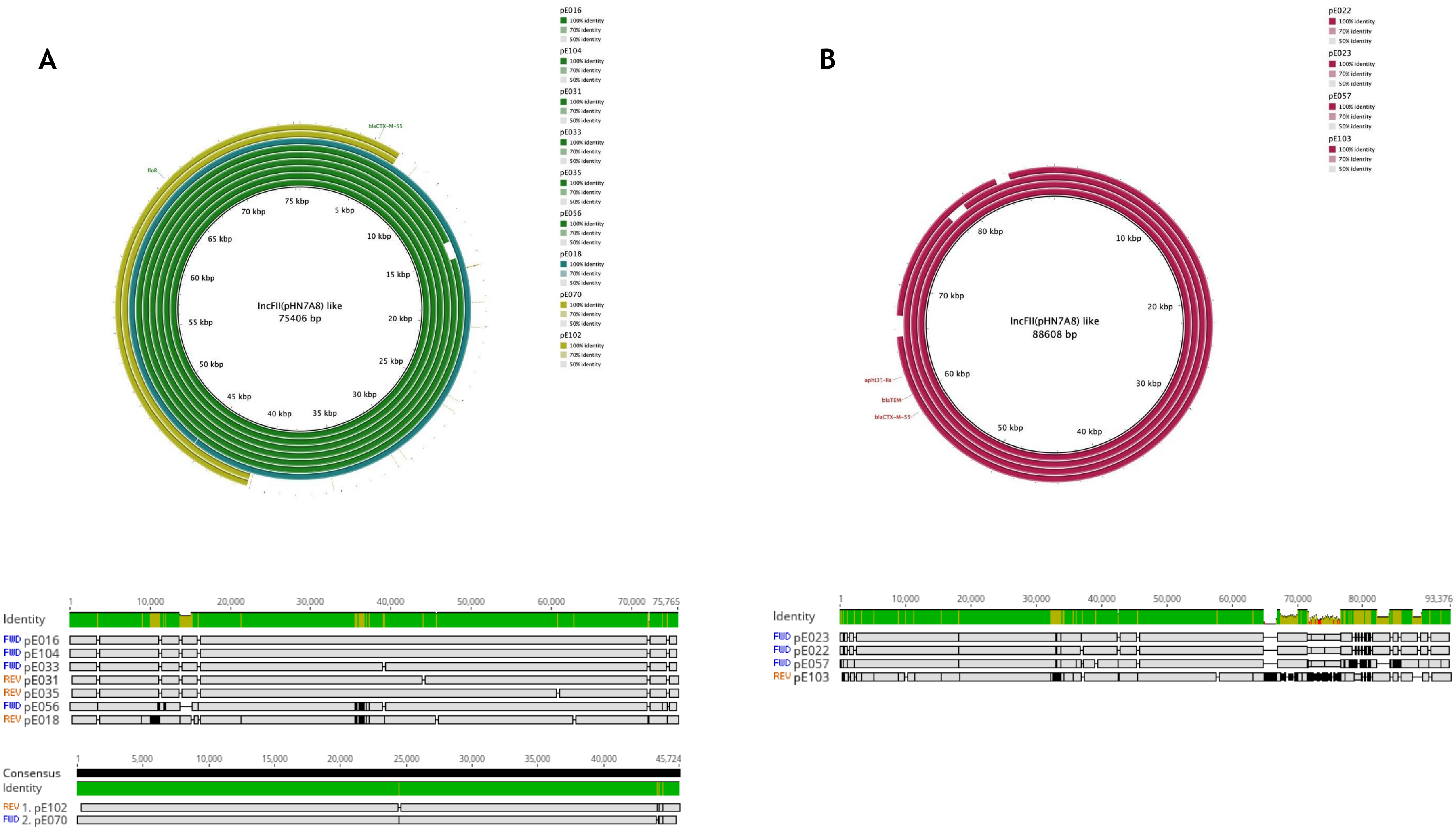

Supplementary Figure 2. The multiple circular and linear sequence alignment of **A:** plasmids IncFII(pHN7A8) harboring *bla*$_{CTX\text{-}M\text{-}55}$ and *ffoR* and **B:** plasmids IncFII(pHN7A8):IncX1 harboring *bla*$_{CTX\text{-}M\text{-}55}$ and *aph(3')-IIa_2*

Supplementary Table I. Plasmid Characterization

Sample	FliC_allele	Plasmid Name	Length (bp)	Replicons	Resistance Genes	pMLST	ST
E033	H21_1	pE033	75405	IncFII(pHN7A8)	*bla*$_{CTX\text{-}M\text{-}55}$; *floR*	FII_33	[F33:A-:B-]
E031	H21_2	pE031	75405	IncFII(pHN7A8)	*bla*$_{CTX\text{-}M\text{-}55}$; *floR*	FII_33	[F33:A-:B-]
E035	H21_3	pE035	75405	IncFII(pHN7A8)	*bla*$_{CTX\text{-}M\text{-}55}$; *floR*	FII_33	[F33:A-:B-]
E070	H21_1	pE070	45463	IncFII(pHN7A8)	*bla*$_{CTX\text{-}M\text{-}55}$; *floR*	FII_33	[F33:A-:B-]
E067	H34_3	pE067_1	131677	IncX1; IncN	(*bla*$_{CTX\text{-}M\text{-}55}$)x3; (*bla*$_{TEM\text{-}141}$)x2; *sul3*; *aph(3')-IIa*; *fosA3*; *aac(3)-IId*	repN_1	Unknown
		pE067_2	127426	IncFIB(AP001918); IncFIC(FII)	*bla*$_{CTX\text{-}M\text{-}55}$; *floR*; *aph(3")-Ia*; *sul3*; *ant(3")-Ia*; *dfrA14*	FII_18; FIC_4; FIB_1	[F18:A-:B1]
E104	H21_1	pE104	75406	IncFII(pHN7A8)	*bla*$_{CTX\text{-}M\text{-}55}$; *floR*	FII_33	[F33:A-:B-]
E103	H26_1	pE103	89363	IncFII(pHN7A8)	*bla*$_{CTX\text{-}M\text{-}55}$; *bla*$_{TEM\text{-}141}$; *aph(3')-IIa*	FII_37*	[F-:A-:B-]
E102	H42_1	pE102	45463	IncFII(pHN7A8)	*bla*$_{CTX\text{-}M\text{-}55}$; *floR*	FII_33	[F33:A-:B-]
E016	H21_1	pE016	75406	IncFII(pHN7A8)	*bla*$_{CTX\text{-}M\text{-}55}$; *floR*	FII_33	[F33:A-:B-]
E018	H42_1	pE018	75437	IncFII(pHN7A8)	*bla*$_{CTX\text{-}M\text{-}55}$; *floR*	FII_33	[F33:A-:B-]
E022	H34_1	pE022	88608	IncFII(pHN7A8); IncX1	*bla*$_{CTX\text{-}M\text{-}55}$; *bla*$_{TEM\text{-}141}$; *aph(3')-IIa*	FII_33	[F33:A-:B-]
E023	H34_2	pE023	88610	IncFII(pHN7A8); IncX1	*bla*$_{CTX\text{-}M\text{-}55}$; *bla*$_{TEM\text{-}141}$; *aph(3')-IIa*	FII_33	[F33:A-:B-]
E056	H21_1	pE056	74056	IncFII(pHN7A8)	*bla*$_{CTX\text{-}M\text{-}55}$; *floR*	FII_33	[F33:A-:B-]
E057	H34_3	pE057	86868	IncFII(pHN7A8); IncX1	*bla*$_{CTX\text{-}M\text{-}55}$; *bla*$_{TEM\text{-}141}$; *aph(3')-IIa*	FII_33	[F33:A-:B-]

Supplementary Table II. Summary of Sequencing and Assembly Statistics

Sequence	Sequence length (bp)	Number of reads	Mean read length (bp)	Mean read quality (bp)	Read length N50 (bp)	Sequencing depth	Coverage Percent
**E016.fasta**	4,715,929	40,484	1,957.2	16.8	6,981	132.54x	100
**E031.fasta**	4,715,929	298,051	1,986.7	16.8	6,623	117.18x	100
**E033.fasta**	4,716,800	915,259	1,825.6	17.1	8,659	323.83x	100
**E035.fasta**	4,702,598	307,903	1,791.4	16.6	6,838	109.85x	100
**E070.fasta**	4,715,928	275,705	2,128.0	17.0	7,797	118.18x	100
**E102.fasta**	5,172,204	787,399	927.0	16.6	6,982	133.85x	100
**E104.fasta**	4,714,593	443,223	1,383.2	17.1	7,872	119.11x	100
**pE016.fasta**	75,406	10,364	17,387.1	16.9	17,871	257.18x	100
**pE018.fasta**	75,437	1,846	14,446.0	16.3	13,988	25.58x	100
**pE022.fasta**	88,608	3,642	13,927.1	16.7	13,393	104.34x	100
**pE023.fasta**	88,61	3,669	15,678.4	16.9	15,474	101.87x	100
**pE031.fasta**	75,405	35,601	17,268.5	17.9	17,383	3366.23x	100
**pE033.fasta**	75,405	55,117	17,234.6	17.9	17,500	4788.45x	100
**pE035.fasta**	75,405	5,368	17,295.2	18.1	17,315	625.62x	100
**pE056.fasta**	74,056	1,032	16,215.8	16.5	16,065	20.02x	100
**pE057.fasta**	86,868	2,824	14,689.0	16.0	13,855	45.32x	100
**pE067_1.fasta**	131,539	7,319	18,193.1	17.5	19,147	319.82x	100
**pE067_2.fasta**	127,431	7,319	18,193.1	17.5	19,147	334.81x	100
**pE070.fasta**	45,463	9,587	18,423.6	18.1	19,437	1946.83x	100
**pE102.fasta**	45,463	76,851	7,069.3	15.9	7,836	348.49x	100
**pE104.fasta**	75,406	14,502	16,640.0	17.9	16,619	1553.54x	100

Note: Sequencing coverage and depth were estimated by aligning FASTQ reads to their corresponding reference FASTA sequences using minimap2 v2.24 in map-ont mode. The resulting SAM alignment files were converted to BAM format, sorted, and indexed using samtools v1.17. Per-base coverage (sequencing depth) was obtained using the samtools depth command, and the average coverage was calculated as the arithmetic mean of the third column (depth) in the resulting file. General read quality and length statistics were computed using NanoStat v1.5.0. The total assembly length and basic sequence statistics were determined using seqkit v2.6.1.
